# Testosterone, cognitive decline and dementia in ageing men

**DOI:** 10.1007/s11154-022-09728-7

**Published:** 2022-05-28

**Authors:** Bu B. Yeap, Leon Flicker

**Affiliations:** 1 Medical School, University of Western Australia, Perth, Australia; 2 Department of Endocrinology and Diabetes, Fiona Stanley Hospital, Perth, Australia; 3 Western Australian Centre for Health and Ageing, University of Western Australia, Perth, Australia; 4 Department of Geriatric Medicine, Royal Perth Hospital, Perth, Australia

**Keywords:** Testosterone, Sex hormone-binding globulin, Cognition, Dementia, Alzheimer’s disease, Male ageing

## Abstract

As men grow older, circulating testosterone concentrations decline, while prevalence of cognitive impairment and dementia increase. Epidemiological studies of middle-aged and older men have demonstrated associations of lower testosterone concentrations with higher prevalence and incidence of cognitive decline and dementia, including Alzheimer’s disease. In observational studies, men with prostate cancer treated by androgen deprivation therapy had a higher risk of dementia. Small intervention studies of testosterone using different measures of cognitive function have provided inconsistent results, with some suggesting improvement. A randomised placebo-controlled trial of one year’s testosterone treatment conducted in 788 men aged ≥ 65 years, baseline testosterone < 9.54 nmol/L, showed an improvement in sexual function, but no improvement in cognitive function. There is a known association between diabetes and dementia risk. A randomised placebo-controlled trial of two year’s testosterone treatment in 1,007 men aged 50–74 years, waist circumference ≥ 95 cm, baseline testosterone ≤ 14 nmol/L, showed an effect of testosterone in reducing type 2 diabetes risk. There were no cognitive endpoints in that trial. Additional research is warranted but at this stage lower testosterone concentrations in ageing men should be regarded as a biomarker rather than a proven therapeutic target for risk reduction of cognitive decline and dementia, including Alzheimer’s disease.

## Testosterone and male ageing

As men grow older, circulating total testosterone concentrations generally decline while medical comorbidities become more prevalent [1–6]. By contrast, concentrations of sex hormone-binding globulin (SHBG), the principal carrier protein in the circulation for testosterone and other sex steroids, increase with age [2, 4–6]. In predominantly middle-aged men, the reduction in total testosterone concentrations largely reflects the impact of obesity and ill-health to reduce activity of the hypothalamic-pituitary–testicular (HPT) axis [3, 4]. This is supported by the finding that in a cohort of healthy men aged 40–69 years, total testosterone concentrations were stable over time, although SHBG increased and cFT declined [5]. However, in older men, total testosterone concentrations decline in parallel with increases in luteinising hormone (LH) concentrations, indicating progressive impairment of Leydig cell function [6, 7]. Free testosterone, the fraction in the circulation not bound to either SHBG or albumin, is commonly calculated from total testosterone and SHBG [8, 9]. Longitudinal declines in calculated free testosterone (cFT) tend to be steeper than the corresponding changes in total testosterone, reflecting the age-related increase in SHBG [2, 4, 5]. Lower testosterone concentrations in ageing men are associated with a range of poorer health outcomes, including cardiovascular events and mortality [10, 11]. A comparable association with dementia risk has been postulated but remains under debate [12–16]. If a causal relationship can be proven, then there may be scope for testosterone to be employed as a preventive or therapeutic intervention.

## Cognitive decline, dementia and Alzheimer’s disease

The convergence of declining circulating testosterone, impairment of cognitive function and increasing diagnoses of dementia, in ageing men, is of interest for a number of reasons. Due to increases in life expectancy in the twentieth century, population structures are maturing world-wide, and increasing proportions of adults in the community will be older [17]. Age is a strong but irreversible risk factor for cognitive decline and incidence of dementia, albeit these are not inevitable consequences of ageing [18]. Deterioration in cognitive function can affect multiple domains of memory, thinking, orientation, comprehension, calculation, learning capacity, language, and judgement. This may be associated with changes in mood, emotional control, behaviour, or motivation, and impact on social functioning and self-care [18]. Risk factors include less education, smoking, obesity, excessive alcohol consumption, traumatic brain injury, hypertension, hearing impairment, depression, air pollution, physical inactivity, diabetes, and low social contact, and preventive strategies are now advocated [19, 20]. Even so, there are 50 million people living with dementia worldwide, with nearly 10 million new cases each year [19]. Dementia due to Alzheimer disease is the most common form of dementia, accounting for 60–70% of cases [18]. This form of dementia is characterised by insidious onset with memory impairment being the initial complaint. Some studies have found a higher prevalence of Alzheimer’s disease in women, generating interest in the possible role of sex hormones to modulate this risk [21, 22]. Population based surveys of people who have been carefully examined with cognitive testing prior to death have clearly demonstrated that the most common neuropathological changes found in dementia due to Alzheimer disease are mixed pathologies, commonly a combination of Alzheimer neuropathology, vascular pathology, age-related TAR DNA binding protein TDP-43 encephalopathy and Lewy body pathology [23, 24]. Recent observations suggest even more pathological mechanisms underly common clinical types of dementia including glial fibrillary acidic protein which is associated with presence of β-amyloid, and phosphorylated tau 231 protein [25–28]. Alzheimer pathology involves extra-neuronal deposition of β-amyloid plaque and intra-neuronal development of neurofibrillary tangles of hyperphosphorylated tau proteins within the central nervous system [24]. Biomarkers of early disease include plasma neurofilament light chain that has also been associated with neurodegeneration from several causes, including in the setting of Alzheimer disease [29, 30]. Treatments aimed at improving neurocognitive function or slowing its decline generally have shown limited efficacy [24, 31, 32]. Recently, considerable attention has been given to interventions which reduce accumulation of β-amyloid, however, clinical benefits have not been conclusively demonstrated [31, 32]. It is possible that hormonal treatments that target two or more of these pathologies may have a specific role in preventing the common age-associated dementia. Thus the possibility that lower testosterone exposure may be involved in the pathophysiology of dementia due to Alzheimer disease is of interest, as interventions to increase testosterone concentrations in men are readily available [33–35].

## Mechanistic studies of testosterone, dementia and Alzheimer’s disease

Experimental studies suggest a role for sex steroids to influence the accumulation of β-amyloid and also to modulate neuronal responses to injury [36]. In murine neuroblastoma and primary neuronal cultures, and in rat hypothalamic cells, treatment with testosterone increased cleavage of the β-amyloid precursor protein to enhance secretion of non-amyloidogenic fragments [37, 38]. In experiments in rat hippocampal neurones, testosterone and estradiol had differential effects on cleavage of tau proteins, and testosterone was more effective in preventing β-amyloid-induced cell death [39–41]. In a transgenic mouse model overexpressing amyloid precursor protein, downregulating aromatase expression increased testosterone and reduced estradiol concentrations, and reduced plaque formation within the brain, inferring a role for testosterone rather than estradiol to protect against Alzheimer’s disease [42]. However, other studies report a role for estrogens and progesterone to protect cultured rat hippocampal neurones against glutamate toxicity [43]. Estradiol (and also estrone and estriol) also appeared to inhibit β-amyloid formation *in vitro* [44].

Of note, in a transgenic mouse model of Alzheimer’s disease, treatment of gonadectomised mice with testosterone prevented the increase in β-amyloid in the subiculum, hippocampus and amygdala, seen in gonadectomised, vehicle-treated mice [45]. In that study dihydrotestosterone had a similar effect, while estradiol prevented β-amyloid accumulation in the hippocampus but only had partial effects in the other two regions. Therefore, inhibition of β-amyloid accumulation may involve both androgen- and estrogen-mediated effects within the brain. In a further report, testosterone treatment in gonadectomised transgenic mice reduced β-amyloid accumulation and improved performance in a hippocampal-dependent task of working memory and attention [46]. Testosterone depletion has also been reported to increase susceptibility to oxidative brain damage in mice [47]. Testosterone and androgen receptor signalling have also been implicated in synaptic formation and plasticity, in the pre-frontal cortex and other areas [48, 49]. However, a study of middle-aged and older rats given a high-fat diet reported relatively subtle effects of testosterone treatment over 12 weeks to prevent diet-induced increases in microglial and astroglial reactivity, with equivocal effects on behavioural outcomes [50]. This illustrates the difficulty of translating results from mechanistic studies based on reducing β-amyloid accumulation to studies demonstrating more clinically-oriented outcomes. The observation that vascular pathology contributes nearly as much to the overall burden of dementia as Alzheimer pathology [23] and the known association of testosterone with vascular risk [10] highlights the potential importance of multimodal effects on overall risk of dementia.

## Observational studies of testosterone, cognitive decline and dementia

### Studies with surrogate endpoints

In a longitudinal study of 514 pairs of twin men aged 63 years at baseline and followed for 10 and 16 years, sex hormones were not associated with measures of cognitive function [51]. However, in that study higher baseline testosterone concentrations were associated with larger hemisphere, frontal lobe and parietal lobe volumes, and smaller left (but not right) occipital lobe volumes, after adjusting for SHBG concentrations. In a smaller study of 40 men aged 57 years at baseline, a higher ratio of testosterone to SHBG and higher total testosterone concentrations measured over 14 years, were associated with higher regional cerebral blood flow assessed by positron emission tomography (PET) [52]. This finding extends an earlier uncontrolled study of seven men aged 58–72 years with sexual symptoms and low cFT concentrations given testosterone treatment, which showed enhanced cerebral perfusion in midbrain and superior frontal gyrus at 3–5 weeks of treatment, and in midbrain and midcingulate gyrus at 12–14 weeks [53]. A post-mortem study found that in men aged 60–79 years, brain testosterone concentrations were lower in men with mild neuropathology or Alzheimer’s disease (N = 7 and N = 22 respectively), compared with neuropathologically normal men (N = 7) [54]. In a study of 118 men who underwent Pittsburgh compound B (PiB)-PET scanning for detection of brain β-amyloid, cFT was not associated with PiB retention [55]. In that study, only in a subset of 24 men with mild cognitive impairment was there an inverse association of cFT with PiB retention. A recent study of 133 men aged 72 years, found that cFT concentration was not associated with presence of cerebral β-amyloid measured using PiB PET-magnetic resonance imaging (MRI), but was associated with hippocampal volume [56]. Thus higher testosterone exposures may be related to more favourable indices of brain volume and regional perfusion, but limited data are available with regards to associations of sex hormones with Alzheimer pathology. The possible implication may be that testosterone might have beneficial effects on the brain through non-Alzheimer pathology-related mechanisms. These contrasting results reflect differences between imaging modalities, and need to consider the inherent limitations of extrapolating results from surrogate measures to longer term clinical outcomes.

### Prospective cohort studies

Longitudinal studies of sex hormones with the outcomes of cognitive decline or incident dementia in middle-aged to older men are summarised (Table 1). In the Baltimore Longitudinal Study of Aging, men with a higher ratio of testosterone to SHBG at baseline performed better on tests of cognitive function, and were less likely to develop Alzheimer’s disease, during extended follow-up (10 and 19 years respectively) [57, 58]. However, in the Honolulu-Asia Asia Study, in which 134 men developed Alzheimer’s disease over a 6-year follow-up, higher baseline estradiol was associated with increased risk, while baseline testosterone was not associated [59]. In the Osteoporotic Fractures In Men Study neither baseline cFT nor baseline calculated free estradiol were associated with changes in cognitive function over 4.5 years [61]. In smaller studies with shorter durations of follow-up, one study found that neither cFT nor estradiol concentrations were associated with dementia risk in men [60], in another higher bioavailable (non-SHBG bound) testosterone concentrations at baseline were associated with lower risk of incident dementia, albeit the actual number of cases was small [62]. A case–control study reported a non-linear association of baseline testosterone with dementia risk, suggesting both lower and higher concentrations might be associated with higher risk [63]. One consideration in observational studies is the extent to which lower testosterone concentrations reflect underlying poorer general health, which may contribute to the outcomes of interest. Analyses were adjusted for potential confounders including age, smoking, BMI and medical comorbidities [57–63], and in some cases also for *APOE* Ɛ4 status [60, 62, 63]. However, residual confounding from unmeasured variables, possibly reflecting poorer general health, remains possible in observational analyses.

**Table 1:** Selected prospective cohort studies which conducted observational analyses of testosterone as the exposure, and incidence of cognitive impairment or dementia (including dementia due to Alzheimer disease) as the outcome

Study author and year	Size (N men)	Age (years)	Follow-up (years)	Summary of results
Moffat et al. [57]	407	50–91	10	Higher ratio of testosterone to SHBG was associated with better scores on visual and verbal memory, visuospatial functioning, visuomotor scanning, and lower rate of longitudinal decline in memory
Moffat et al. [58]	574	32–87	19	54 men developed dementia due to Alzheimer disease^a^. Increased ratio of testosterone to SHBG was associated with decreased risk (hazard ratio 0.74 per 10 nmol/mol increase)
Geerlings et al. [59]	2,974	71–93	6	134 men developed dementia of Alzheimer’s type^a^. Testosterone was not associated with risk of dementia, higher estradiol concentrations were associated with risk of Alzheimer’s disease (hazard ratio 1.25 per 1 SD increase)
Ravaglia et al. [60]	376	≥ 65	3.8	39 men developed dementia (23 dementia of Alzheimer’s type, 12 vascular dementia)^a^. Neither cFT nor estradiol concentrations were associated with risk of dementia
LeBlanc et al. [61]	1,022	≥ 65	4.5	No association of baseline cFT or calculated free estradiol with change in cognition. Higher SHBG was associated with increased risk of cognitive decline (executive function and motor speed, general cognition)
Chu et al. [62]	155	≥ 55	1	10 men developed dementia.^a^ Higher bioavailable testosterone (measured using ammonium sulphate precipitation) associated lower risk of Alzheimer’s disease at 1 year
Carcaillon et al. [63]	503	≥ 65	4	105 men who developed incident dementia^a^, and random sample of 413 men as controls. Non-linear association of dementia with baseline testosterone (hazard ratio lower tertile 2.33, P = 0.026, upper tertile 1.9, P = 0.126, vs middle tertile). Risk of dementia associated with lower bioavailable testosterone was greater in men aged ≥ 80 vs men aged < 80 years
Ford et al. [14]	4,069	71–88	10.5	499 men developed dementia^b^. Lower baseline testosterone was associated with higher risk of incident dementia (hazard ratio 1.14 per 1 SD decrease), as was lower cFT (hazard ratio 1.18 per 1 SD decrease). Lower estradiol was associated with higher risk of incident dementia (hazard ratio 1.11 per 1 SD decrease) but SHBG was not associated
Marriott et al. [13]	159,411	40–69	7	826 men developed dementia, of which 288 were classified as having Alzheimer’s disease^b^. Lower testosterone concentrations were associated with higher incidence of dementia (overall trend P = 0.001, hazard ratio 1.43 for lowest vs highest quintile), and Alzheimer’s disease (overall trend P = 0.017, hazard ratio 1.80 for lowest vs highest quintile). Lower SHBG was associated with lower incidence of dementia and Alzheimer’s disease (P = < 0.001, hazard ratio 0.66; P = 0.012, hazard ratio 0.53 for lowest vs highest quintile, respectively)

Multiple criteria were used for ascertainment of dementia outcomes including Diagnostic and Statistical Manual of the American Psychiatric Association (DSM), International Classification of Disease of the World Health Organization (ICD) and the National Institute of Neurological and Communicative Disorders and Stroke and the Alzheimer's Disease and Related Disorders Association (NINCDS-ADRDA) Alzheimer’s criteria

^a^cognitive assessment undertaken

^b^dementia diagnosis based on hospital morbidity and other health registry data

In the Health In Men Study (HIMS), of 4,069 men aged 71–88 years, 499 developed dementia during a median of 10.5 years follow-up [14]. In that study, after adjusting for age and other comorbidities, lower testosterone, cFT and estradiol concentrations at baseline were all associated with higher risk of incident dementia during follow-up, while SHBG was not associated. The effect of older age was evident: mean age at baseline of the 499 men who developed dementia was 78.7 years, compared with 76.8 years for men who remained free of dementia, and there was a 15% increased risk of dementia per year of increased age. In comparison the risk of dementia increased by 11% per 1 standard deviation lower baseline total testosterone concentration [14]. A strength of HIMS was the measurement of sex hormones using mass spectrometry and the large number of outcome events observed (consistent with the size of the cohort and the age of the men, as well as duration of follow-up). Dementia outcomes were ascertained using registry data and were not independently adjudicated. The largest prospective cohort study to report associations of testosterone and SHBG with dementia outcomes is the United Kingdom (UK) Biobank, in which 159,411 community-dwelling men, aged 50–73 years, were followed for 7 years, with 826 men developing dementia of whom 288 were classified as having dementia due to Alzheimer disease [13]. In that study, median age at baseline was 61 years. After adjustment for sociodemographic, lifestyle and medical factors and medications use, there were contrasting associations of testosterone and SHBG. Men with lower testosterone concentrations had a higher incidence of dementia, and of dementia due to Alzheimer disease. Those in the lowest quintile of total testosterone concentrations had a 43% increased risk of developing dementia, and an 80% increased risk of dementia due to Alzheimer disease, compared with men in the highest quintile. Lower cFT was similarly associated with higher risk of dementia and AD. By contrast, men with lower SHBG concentrations had a lower incidence of dementia, and of dementia due to Alzheimer disease [13]. Although this cohort included middle-aged as well as older men, then median age at diagnosis of incident dementia was 70 years, and 82% of men were diagnosed at age ≥ 65 years. UK Biobank also utilised registry data to ascertain diagnoses of dementia and dementia due to Alzheimer disease, using an algorithm validated against clinical expert adjudication of full-text medical records [64, 65]. Thus, two large population-based cohort studies, of middle-aged to older men in UK Biobank, and older men in HIMS, found consistent associations of lower baseline testosterone concentrations with higher incidence of dementia, and of dementia due to Alzheimer disease [13, 14]. Higher SHBG may be associated with both cognitive decline in older men [61], and higher incidence of dementia and Alzheimer’s disease in middle-aged to older men [13]. Thus in observational studies, testosterone concentrations are inversely, and SHBG concentrations directly, associated with risk of developing dementia and Alzheimer’s disease.

## Studies of men undergoing androgen deprivation therapy for prostate cancer

Two small observational studies reported increases in β-amyloid concentrations in men following androgen deprivation therapy (ADT) for treatment of prostate cancer [66, 67]. In other small observational studies of men undergoing ADT for treatment of prostate cancer, inconsistent effects on cognitive function have been observed [67–71]. These studies were limited by their relatively small size and lack of control groups, with some studies associating ADT with poorer performance in selected measures of cognitive function, while other studies showed no associations or even better performance in other tests.

In a study comparing 15 men receiving ADT and 15 not receiving ADT, results of cognitive function tests were similar between groups at 6 months [72]. However, functional MRI studies showed subtle differences in medial prefrontal cortical activation, and connectivity between the medial prefrontal cortex and other regions involved with cognitive control between groups [72]. Other studies have not shown consistent effects of ADT on cognition [73, 74]. However, in a trial involving 82 men with prostate cancer, 24 of 50 men randomised to ADT had a decline in one or more cognitive tests at 6 months, whereas none of 15 men randomised to close observation showed a decline in any test performance [75]. Therefore, ADT may have an adverse effect on cognition, most likely visuospatial abilities and executive functioning, but the evidence is very limited and not wholly consistent [76, 77]. A systematic review and meta-analysis of 14 studies with 417 patients with prostate cancer treated with ADT, associated ADT with worse performance on visuomotor tasks, but not other cognitive domains, compared to noncancer control groups [78]. A recent systematic review of 31 studies found that 16 did not show a negative effect of ADT on cognition, whereas 11 studies reported a negative effect on cognitive function and 4 were inconclusive [79]. Therefore, ADT may have adverse effects on cognitive performance, but this is not invariable and may be limited to specific domains.

Of note, two recent large registry studies have associated ADT for prostate cancer with higher risk of dementia [80, 81]. In a study of 23,651 men with prostate cancer median age 73 years, 1,525 were diagnosed with incident dementia during median follow-up of 3.5 years. Men receiving antiandrogen monotherapy had a higher risk of dementia and Alzheimer’s disease compared to men who did not receive ADT [80]. In that study the risk of dementia in men receiving GnRH agonist treatment, or those who underwent orchidectomy, was similar to those who received no ADT. In a study of 13,570 men with prostate cancer aged ≥ 50 years, 317 were diagnosed with dementia after median 7.0 years follow-up [81]. Cumulative ADT exposure was associated with dementia risk. A meta-analysis of seven cohort studies including 90,543 men with prostate cancer (38,307 exposed to ADT and 52,236 non-exposed), associated ADT with higher incidence of subsequent dementia [82]. Another meta-analysis of seven studies including 50,541 individuals showed an increased risk of dementia in ADT users [83]. Important factors to consider in the context of variable findings include differences in cognitive assessments or categorisation of dementia, the type, duration and intensity of ADT, analytical strategies to minimise bias, and polygenic or multifactorial determinants of dementia risk.

## Randomised trials of testosterone with cognitive function endpoints

### Trials in general populations of men

A large number of clinical trials with relatively small numbers of participants, have been conducted to investigate the effects of testosterone, for generally limited durations of intervention, on various cognitive function tests as endpoints in relatively small numbers of participants (Table 2). Earlier studies included 15 to 88 participants, used transdermal, oral or intramuscular formulations of testosterone, and were conducted mainly in older men from the general population [84–94, 96]. Three studies of 12 months found no effect of testosterone on cognitive abilities [85, 88, 89]. In one study of 1.5 months duration, inducing hypogonadism in groups of younger and older men, did not appear to modify cognition [96]. Five studies reported improvements in tests of visuospatial cognition: in three studies with durations ranging from 1.5 to 5 months there were no effects on other domains of cognitive function [84, 86, 90], in two studies with duration of 1.5 months there were also concomitant improvements in verbal memory [91, 94]. One two-month study reported decreased performance in tests of verbal memory in levonorgestrel-treated men, but improved selective attention in men receiving testosterone and levonorgestrel [87]. One nine-month study reported a decrease in verbal memory with testosterone treatment [92]. A 36 month study found no difference in multiple cognitive tests, except for improved verbal memory in older men treated with testosterone and finasteride [93]. Therefore, some but not all of these earlier and smaller trials reported a possible benefit of testosterone intervention primarily on spatial cognition after shorter durations of intervention. However, there were inconsistent results for verbal memory, and several longer duration trials did not find evidence of benefit.

**Table 2:** Selected randomised, placebo-controlled studies of testosterone treatment in men with outcomes related to memory and other measures of cognitive performance. N denotes men in active and placebo arms

Study author and year	Eligibility criteria	Formulation of androgen	N active	N placebo	Duration (months)	Result
Janowsky et al. [84]	60–75 years	Transdermal T 15 mg scrotal patch daily	27	29	3	Enhanced spatial cognition; no effect on verbal memory, dexterity, or cognitive flexibility
Sih et al. [85]	≥ 50 years, non-SHBG bound T ≤ 2.1 nmol/L	IM T 200 mg fortnightly	17	15	12	No effect on memory, recall or verbal fluency
Cherrier et al. [86]	50–80 years	IM T 100 mg weekly	15	13	1.5	Improved spatial memory and ability, and verbal memory; no effect on attention or verbal fluency
Cherrier et al. [87]	21–46 years	IM T 100 mg weekly ± oral LN 125 µg daily	32		2	Decreased performance in tests of verbal memory in LN-treated group, improved selective attention in T + LN group
Kenny et al. [88]	≥ 65 years, non-SHBG bound T ≤ 4.4 nmol/L	Transdermal T 5 mg patch daily	24	40	12	No difference in cognitive test results between groups
Haren et al. [89]	≥ 60 years, T/SHBG 0.3–0.5, T > 8 nmol/L	Oral T 80 mg bd	39	37	12	No difference in visuomotor tracking and visuospatial ability
Gray et al. [90]	60–75 years	GnRH + IM T 25, 50, 125, 300, 600 mg weekly	60		5	Differences in visuospatial cognition across treatment groups, with highest scores in men on highest dose (600 mg/week)
Cherrier et al. [91]	50–90 years	IM T 100 mg weekly ± oral AN 1 mg daily	60		1.5	Improved spatial memory in T and T + AN groups, improved verbal memory in T group only
Maki et al. [92]	66–86 years	IM T 200 mg fortnightly	15		9*	Decreased verbal memory
Vaughan et al. [93]	65–83 years, T < 12.1 nmol/L	IM T 200 mg fortnightly ± oral F 5 mg daily	69		36	No differences in multiple cognitive tests; except for improved verbal memory with T + F
Cherrier et al. [94]	50–90 years	IM T 50, 100 or 200 mg weekly	57		1.5	Improved verbal and spatial memory associated with moderate increases in T, not with low or large increases
Emmelot-Vonk et al. [95]	60–80 years, T < 13.7 nmol/L	Oral T 80 mg bd	113	110	6	No differences in verbal memory, perceptual speed, attention, or visuospatial performance
Young et al. [96]	25–35 years, 60–80 years	GnRH + transdermal T gel 100 or 75 mg ± AN 1 mg/d oral	26, 62		1.5	No effect on measures of executive function, memory and spatial cognition
Huang et al. [97]	≥ 60 years, T 3.5–13.9 nmol/L or free T < 173 pmol/L	Transdermal T gel 75 mg/d	140	140	36	No differences in visuospatial ability, verbal fluency, verbal memory, manual dexterity, attention or executive function
Gregori et al. [98]	≥ 65 years, BMI ≥ 30 kg/m^2^, frailty, T < 10.4 nmol/L	Background lifestyle intervention, transdermal T gel 40.5 mg/d	42	41	6	Improved visuospatial performance, attention, verbal memory, and global cognition scores

*IM* intramuscular, *T* testosterone, *LN* levonorgestrel, *GnRH* gonadotrophin-releasing hormone, *AN* anastrazole, *F* finasteride, *BMI* body mass index

^*^ cross-over design

Two larger and more recent clinical trials merit particular attention [95, 97]. Emmelot-Vonk et al. randomised 237 healthy men aged 60–80 years with baseline total testosterone concentrations < 13.7 nmol/L, to oral testosterone or matching placebo for 6 months, with 223 included in the primary analysis [95]. In that study testosterone treatment reduced fat mass and increased lean mass, but there were no differences between groups for visuospatial performance, perceptual speed, attention, or verbal memory. The Testosterone Effects on Atherosclerosis in Aging Men (TEAAM) trial randomised 308 men aged ≥ 60 years who had baseline total testosterone concentrations of 3.4–13.9 nmol/L, or a calculated free testosterone value of < 173 pmol/L, to transdermal testosterone or matching placebo, for 3 years [97]. In the TEAMM trial, 140 men in each group were included in the final analysis, finding no benefit of testosterone treatment for visuospatial ability, verbal fluency, verbal memory, manual dexterity, attention or executive function. Therefore, two major testosterone randomised controlled trials in the general population of middle-aged to older men with low-normal testosterone concentrations, one of 6 months oral testosterone, the other of 36 months transdermal testosterone, have not found a benefit of testosterone intervention on cognition.

Of note, a recent secondary analysis of the Lifestyle Intervention and Testosterone Replacement in Obese Seniors (LITROS) trial, examined the effect of transdermal testosterone compared to placebo, on a background of an intensive weight management and exercise program [98]. Participants in LITROS were older men (≥ 65 years) who were obese (BMI ≥ 30 kg/m^2^), had baseline total testosterone concentrations < 10.4 nmol/L and had evidence of mild to moderate physical frailty. The intensive lifestyle intervention consisted of a weight management program (aiming to achieve 10% weight loss at six months) and exercise training, provided to all trial participants, on top of which they were randomised to transdermal testosterone or matching placebo. In LITROS, testosterone treatment resulted in improved visuospatial performance, attention, verbal memory, and global cognition scores, compared to placebo [98]. This was a smaller study compared with the Emmelot-Vonk et al. and TEAAM trials [95, 97]. Nevertheless the findings are intriguing, suggesting that testosterone treatment in obese older men, applied in conjunction with an intensive lifestyle intervention, may result in improvement across a range of cognitive measures in a relatively short space of time. It is known that exercise may have cognitive benefits for people through the lifecourse [99].

### Trials in men with cognitive impairment or dementia

The above studies were conducted in men from the general population, who were not selected for the presence of cognitive impairment at baseline [84–98]. Therefore, the results may have been influenced by the relative lack of cognitive vulnerability of the participating men. Intervention studies in men at greater risk for cognitive decline or with established dementia, may yield different results compared to low-risk men with robust cognitive function [100–103]. Several interventional studies of testosterone have been conducted targeting groups of men with cognitive impairment as summarised (Table 3).

**Table 3:** Selected randomised, placebo-controlled studies of testosterone treatment in men with mild cognitive impairment (MCI) or with Alzheimer’s disease (AD), with outcomes of cognitive performance. N denotes total number of men randomised

Study author and year	Eligibility criteria	Formulation of androgen	N active	N placebo	Duration (months)	Results
Tan and Pu [103]	68–80 years, newly diagnosed probable Alzheimer’s disease, T < 7 nmol/L	IM T 200 mg fortnightly	5	5	12	Improved general cognition and visuospatial ability
Kenny et al. [104]	≥ 65 years, MCI, non-SHBG bound T ≤ 4.4 nmol/L	IM T 200 mg every 3 weeks	6	5	3	No difference in cognitive test results between groups
Cherrier et al. [105]	63–85 years, Alzheimer’s disease (n = 15) or MCI (n = 17)	IM T 100 mg weekly	AD = 9, MCI = 10	AD = 6, MCI = 7	1.5	Improved spatial memory and ability, and verbal memory; no differences in verbal fluency or attention
Lu et al. [106]	Men with probable Alzheimer’s disease (n = 16) and healthy controls (n = 22)	Transdermal T gel 75 mg daily	AD = 9, controls = 14	AD = 9, controls = 15	6	Trend to improvement in visuospatial function in men with Alzheimer’s disease, no difference in verbal memory
Wahjoepramono et al. [107]	≥ 50 years, T 10.4–20.8 nmol/L, with subjective memory complaints	Transdermal T cream 50 mg daily	22, 22		12*	Improvement in global cognition score
Resnick et al. [108]	T Trials substudy. Men ≥ 65 years, T < 9.54 nmol/L, and age-associated memory impairment	Transdermal T gel 50 mg/d	247	246	12	No effect of testosterone on tests of verbal and visual memory, executive function, or spatial ability, at either 6 or 12 months

*IM* intramuscular, *T* testosterone

^*^ crossover design

Several earlier and relatively small trials in men with mild cognitive impairment or probable Alzheimer disease, reported inconsistent results [103–106]. One small trial in 10 men with newly diagnosed Alzheimer’s disease of fortnightly intramuscular testosterone treatment for 12 months, reported improved general cognition and visuospatial ability [103]. Another trial in 11 men with mild cognitive impairment (MCI) of intramuscular testosterone given every 3 weeks, over 3 months, found no difference in cognitive test results [104]. Two larger trials were more encouraging. One trial in 32 men with either MCI or Alzheimer’s disease, of weekly intramuscular testosterone treatment over 1.5 months, reported improvement in spatial ability and verbal memory [105]. Another using transdermal testosterone over 6 months in 16 men with Alzheimer’s disease, reported a trend to improvement in visuospatial function, but no difference in verbal memory [106]. In another study, 22 men were randomly allocated to testosterone treatment for 24 weeks, and after a 4 week washout crossed over to placebo for 24 weeks, with another 22 men randomly allocated to placebo followed by testosterone [107]. In that study, testosterone treatment was associated with improvement in a global cognition test score. Overall, the findings of these studies should be regarded as being suggestive at best, given the relatively small sample sizes involved [103–107].

The Testosterone Trials (T Trials) recruited 788 men aged ≥ 65 years, with baseline total testosterone < 9.54 nmol/L, and symptoms related to sexual function, physical function, and/or vitality into a series of co-ordinated trials with a 12-month intervention, to test different outcomes [109]. In the Cognition Trial, a subgroup of 493 men met the criteria for age-associated memory impairment (subjective memory complaints and/or objective memory impairment), and were the primary analysis cohort for a series of cognitive endpoints (Table 3) [108]. When the 247 testosterone-treated men were compared to the 246 placebo recipients, there was no effect of testosterone on tests of verbal and visual memory, executive function, or spatial ability, at either 6 or 12 months. The primary outcome of improvement in delayed paragraph recall was not met in the analysis of the Cognition Trial cohort, nor in the analysis of the whole T Trials cohort. However, an exploratory analyses of the entire T Trials cohort found a small improvement in executive function in testosterone-treated men, a secondary outcome [108]. Therefore, the Cognition Trial overall showed a neutral effect of one year’s treatment with transdermal testosterone on cognitive function [110].

These findings highlight the challenges in proving causality. Large, well-powered epidemiological studies adjusting for potential confounders show middle-aged and older men with lower testosterone concentrations have a higher risk of being diagnosed with dementia [13, 14]. Some smaller clinical trials, often using intramuscular injections of testosterone, suggest a benefit of testosterone intervention on specific measures of cognitive function, but other trials have shown no benefit [84–94, 96, 103–107]. Several larger clinical trials using oral or transdermal testosterone have not shown a benefit of testosterone treatment on cognitive function [95, 97, 108]. We hypothesise that epidemiological studies reflect an extended period of exposure to differences in circulating testosterone concentrations, resulting in subtle but cumulative effects on cognition, in a manner not replicated in clinical trials of relatively shorter duration. It is possible that the mechanisms of any putative testosterone-induced benefits may affect multiple different pathologies associated with cognitive decline in older people. It is possible that larger and longer clinical trials may be required, and that route of administration of testosterone and differences in pharmacokinetics may be relevant [34]. It is also possible that combining testosterone with a lifestyle intervention, may provide a more informative clinical trial strategy [98, 111, 112].

The *APOE* Ɛ4 allele is a key genetic risk factor for late-onset Alzheimer’s disease, and an interaction between free testosterone and *APOE* Ɛ4 genotype has been reported [113]. Whether future clinical trials would benefit from incorporating consideration of *APOE* Ɛ4 genotype, or other genetic markers associated with Alzheimer’s disease, into the study design remains to be determined. Genetic polymorphisms associated with testosterone concentrations have been identified, but a recent Mendelian randomisation analysis of UK Biobank men did not find any association between polymorphisms associated with cFT and dementia risk [114, 115].

## Diabetes and dementia

### Diabetes as a risk factor for dementia

Diabetes mellitus is a recognised risk predictor for the development of dementia and dementia due to Alzheimer disease [20, 116–118]. Type 2 diabetes accounts for the large majority of cases of diabetes, and shares risk factors with dementia itself, including overweight/obesity and physical inactivity [116]. Therefore, it is plausible that addressing such risk factors and reducing the incidence of diabetes may reduce the subsequent risk of dementia. Of note, there is a bidirectional association of obesity with lower testosterone concentrations in middle-aged to older men [3, 4]. Men with lower testosterone concentrations are more likely to have or to develop metabolic syndrome or type 2 diabetes [119, 120]. In conjunction with the known associations of lower testosterone concentrations with risk of dementia (as discussed earlier), it has been postulated that testosterone may play some role in the interaction between excess adiposity, diabetes and dementia [121].

Intensive lifestyle interventions aimed at reducing excess weight with a combination of dietary changes and increasing physical activity levels are effective at preventing type 2 diabetes [122, 123]. Exercise is recommended as part of the management of type 2 diabetes, and may help to ameliorate dementia risk in that setting [124, 125]. Therefore, lifestyle measures which prevent or help manage type 2 diabetes may overlap to some degree with lifestyle measures to reduce future risk of dementia [19, 20]. Whether such lifestyle interventions combined with testosterone treatment might protect against both type 2 diabetes and cognitive decline, remains unclear.

### Testosterone prevents or reverses type 2 diabetes in high-risk men

Testosterone for the Prevention of Type 2 Diabetes Mellitus in high-risk men (T4DM) recruited 1,007 men aged 50–74 years, with waist circumference ≥ 95 cm, baseline testosterone concentration ≤ 14 nmol/L and either impaired glucose tolerance or newly diagnosed type 2 diabetes (oral glucose tolerance test [OGTT] 2-h glucose concentration 7.8–11.0 mmol/L or ≤ 15 mmol/L respectively) to a two-year intervention with three-monthly intramuscular testosterone undecanoate versus placebo on a background of a Weight Watchers lifestyle intervention [126, 127]. After two years, type 2 diabetes was present in 87/413 (21%) of men in the placebo group, and 55/443 (12%) men in the testosterone group (relative risk 0.59, 95% confidence interval 0.43–0.80, p = 0.0007) [127]. Testosterone treated men gained on average 0.39 kg of muscle mass and lost 4.6 kg of fat, whereas placebo treated men lost 1.3 kg of muscle mass and 1.9 kg of fat. T4DM is the largest testosterone randomised controlled trial completed to date, with an unequivocal result showing a beneficial effect of testosterone treatment, in the setting of a background lifestyle intervention, to prevent or revert type 2 diabetes in men at high risk. Thus testosterone pharmacotherapy is an option whose benefits and risks may be discussed, and compared with other lifestyle-based or pharmacological interventions [128]. However, T4DM did not include any cognition-related endpoints. Therefore, testosterone treatment in conjunction with lifestyle intervention, addresses two key factors predisposing to dementia, namely obesity and diabetes, but its effect on dementia risk remains unproven.

## Clinical implications and considerations for future testosterone intervention trials

Testosterone exerts systemic effects across multiple tissues [129]. The T Trials sub-studies showed that testosterone treatment improved anaemia, and increased volumetric bone mineral density [130, 131]. Similarly, in T4DM, testosterone treatment improved sexual function, and also improved volumetric bone mineral density predominantly via effects on cortical bone [127, 132]. However, in the T Trials cardiovascular sub-study of 73 testosterone-treated men and 65 men receiving placebo, daily transdermal testosterone treatment was associated with an increase in non-calcified coronary atheromatous plaque volume, but not in coronary calcium score [133]. However, in that sub-study the groups were unbalanced, with men in the placebo group having substantially more plaque at baseline and at the end of the study, posing a challenge when interpreting the results [110]. Further studies to elucidate the effect of testosterone treatment on progression of coronary atheroma and more broadly on cardiovascular risk are required [10, 110]. As vascular pathology is the second commonest pathology found in older people with dementia this has major implications on the risk for dementia.

A United States Food and Drug Administration mandated cardiovascular safety study of daily transdermal testosterone, A Study to Evaluate the Effect of Testosterone Replacement Therapy on the Incidence of Major Adverse Cardiovascular Events (MACE) and Efficacy Measures in Hypogonadal Men (TRAVERSE, NCT03518034), aims to recruit 6,000 men aged 45–80 years who have evidence of or are at increased risk for cardiovascular disease [134]. This study commenced in May 2018, with planned completion in June 2022, with the primary outcome of MACE, secondary outcomes of cardiovascular and prostate safety, and other outcomes of sexual activity, remission of persistent depressive disorder, bone fractures, correction of anaemia, and progression from pre-diabetes to diabetes [134]. Therefore, when completed this study should provide important information on the cardiovascular safety of testosterone in men at high risk of or with prevalent cardiovascular disease. It may confirm other findings from T Trials and T4DM, but will not address the issue of cognitive decline. A future large randomised trial of testosterone with an extended duration of intervention and cognition-related endpoints would be needed. Potential benefits may result from direct effects of testosterone on pathophysiological processes underlying development of dementia and/or indirectly via beneficial effects of testosterone on body composition and metabolism.

## Summary and conclusions

The convergence of population ageing with increasing rates of dementia represents a major global public health challenge. Understanding the relationships between male ageing, declining circulating testosterone concentrations, and increasing cognitive impairment and dementia, may provide important insights into novel preventive strategies. Mechanistic studies indicate that testosterone may have protective effects within the brain, slowing the development of the multiple pathologies found in men with dementia due to Alzheimer disease. Epidemiological studies of middle-aged and older men associate lower testosterone concentrations with higher prevalence and incidence of cognitive decline and dementia. Observational studies of men with prostate cancer have associated ADT with decrements in cognitive function and higher risk of dementia. However, evidence for causality remains elusive.

Smaller testosterone intervention studies using different measures of cognitive function have yielded inconsistent results, with some studies suggesting improvement. The T Trials Cognition Study did not show a benefit of one year of transdermal testosterone treatment on cognition. Diabetes predisposes to dementia, and T4DM showed that two years treatment with intramuscular testosterone undecanoate on a background of lifestyle intervention, prevented or reverted diabetes in men with impaired glucose tolerance or newly diagnosed type 2 diabetes. T4DM did not assess cognitive function as an endpoint, but it does point the way to future studies, in which testosterone treatment could be combined with lifestyle intervention to assess the effects on cognitive function.

Thus additional research is warranted, pending which lower testosterone concentrations in ageing men should be regarded as a biomarker rather than a proven therapeutic target for risk reduction of cognitive decline and dementia, including dementia due to Alzheimer disease.

## Abbreviations

Androgen deprivation therapy: ADT
Body mass index: BMI
Calculated free testosterone: cFT
Gonadotrophin-releasing hormone: GnRH
Health In Men Study: HIMS
Hypothalamic-pituitary-testicular: HPT
Lifestyle Intervention and Testosterone Replacement in Obese Seniors: LITROS
Luteinising hormone: LH
Magnetic resonance imaging: MRI
Major adverse cardiovascular events: MACE
Mild cognitive impairment: MCI
Oral glucose tolerance test: OGTT
Pittsburgh compound B: PiB
Positron emission tomography: PET
Sex hormone-binding globulin: SHBG
Testosterone Effects on Atherosclerosis in Aging Men: TEAAM
Testosterone for the Prevention of Type 2 Diabetes Mellitus: T4DM
The Testosterone Trials: T Trials
Transactive response DNA binding protein of 43 kDa: TDP-43
United Kingdom Biobank: UK Biobank
